# Increased expression of p16 in both oral and genital lichen planus

**DOI:** 10.4317/medoral.22432

**Published:** 2018-06-21

**Authors:** Karin Danielsson, Joakim Olah, Reza Zohori-Zangeneh, Elisabet Nylander, Majid Ebrahimi

**Affiliations:** 1 Department of Odontology, Umeå University, SE – 901 85 Umeå, Sweden; 2 Umeå University. Dental School SE – 901 85 Umeå, Sweden; 3 Dept. of Public health and Clinical Medicine, Dermatology and Venereology, Umeå University, Umeå, Sweden

## Abstract

**Background:**

Lichen Planus, LP, is an inflammatory disease of possible autoimmune origin affecting mainly oral and genital mucosa and skin. According to the WHO oral LP is considered a potentially malignant disorders. The p16 tumour suppressor protein can act as an inhibitor of cyclin dependent kinases 4 and 6 and thus down regulate cell cycle progression. Since the discovery of p16 several studies have evaluated its expression in various forms of human cancers. The aim of this study was to evaluate and compare the expression of p16 in oral and genital LP and corresponding healthy mucosa.

**Material and Methods:**

A total of 76 cases of oral LP (OLP), 34 cases of genital LP (GLP), 12 cases of healthy oral and 9 cases of healthy genital mucosa were analysed by the use of immunohistochemistry.

**Results:**

Data showed p16 to be highly expressed in both oral and genital LP, higher than in oral (*p*=0.000), and genital controls (*p*=0.002).

**Conclusions:**

Results suggest that the over-expression of p16 seen in LP play a part in the histopathology of the disease.

** Key words:**p16, inflammation, oral, genital, lichen planus, malignant risk.

## Introduction

Lichen planus (LP) is a chronic inflammatory disease affecting skin and mucosa. The origin of the disease is unknown but autoimmunity has been suggested to play a part in the pathobiology (1-3). The disease is thought to be mediated by cytotoxic T-cells acting against the oral epithelium (4). A recent study showed that more than 50% of oral LP (OLP) patients also suffer from genital LP (GLP) and that approximately 1/3 of OLP patients also have skin lesions (5). GLP lesions are usually symptomatic with various degrees of pain and burning sensations. In contrast to OLP, which is classified as a potentially malignant disorder by WHO (6) there is little evidence for malignant transformation of GLP. The malignant potential of OLP has, however, been extensively debated. In a review article from 1999, van der Meij concluded that the rate of malignant transformation for OLP should be considered lower than 0.2%, and that more clear and universally accepted diagnostic criteria are needed (7). However, in several studies, a malignification rate of 0.5-2% is reported (8). Such a rate is, however, considered doubtful as it would make OLP the cause of oral cancer in different parts of the world (7).

The p16 protein is the product of the CDKN2 gene located on chromosome 9p21. It plays a crucial role in regulation of the cell cycle (9). p16 prevents the association of CDK4/CDK6 with cyclin D which in turn prevents phosphorylation of important substrates essential for transit through the G1 phase of the cell cycle, resulting in inhibition of cell proliferation (9). Over-expression of p16 has been seen in 13% to 50% of oral squamous cell carcinoma (4,10).

There are so far only a few studies on the expression of p16 in OLP, and results vary with expression rates between 27% up to almost 75% depending on quantification techniques and choice of cut off (4,11-13). Some of these studies have suggested a positive role for p16 in detection of oral dysplasia and also a role in progression to oral squamous cell carcinoma (13,14), whereas other studies claim that p16 cannot be used as a reliable parameter for identification of malignant transformation of OLP (12,15). In contrast to OLP, which is classified, as a premalignant condition there is little evidence of malignant transformation of erosive GLP. There is also an ongoing discussion whether OLP and GLP are the same disease appearing in different mucosal sites.To shed light on this issue we here included OLP and GLP as well as corresponding normal tissue in a study of the expression of the cell cycle regulator p16. The aim of this study was to evaluate and compare the expression of p16 in oral and genital LP and corresponding healthy mucosa.

## Material and Methods

-Patient material

Biopsies from 79 patients diagnosed with OLP and 24 patients with genital LP were retrieved from the archive at Clinical Pathology, Umeå University. The diagnosis of OLP and GLP was both clinically and histologically verified. All cases were in an active state, histologically showing a well-defined inflammatory infiltrate. The OLP group consisted of 53 females (67%) and 26 males (33%) and of the GLP patients 18 were females (75%) and 6 males (25%). The OLP group had an age range of 21-89 years with a mean age of 57 and the GLP group an age range of 33-84 years with a mean age of 51 years ( Table 1). Of 79 OLP samples 57 were collected from buccal mucosa, 11 from gingiva, 3 from tongue, 2 from palate and remaining 6 from other sites.

**Table 1:**
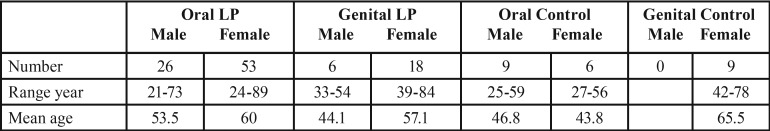
Data on patients and controls (OC = Oral Control and GC= Genital Control) regarding gender and age.

Fifteen biopsies from normal healthy oral mucosa and nine biopsies from normal healthy genital mucosa were also collected and used as control group. All together a total of 127 biopsies were included in the study ( Table 1). The study was approved by the Ethical Review Board at Umeå University (dnr 2013-252-32M).

-Immunohistochemistry

Five µm sections were cut from formalin fixed paraffin embedded samples. Sections were dewaxed, rehydrated by standard procedures and subjected to boiling in 10 mM citrate buffer, pH 6.0 for 15 min using a microwave oven for antigen retrieval.

The antibody used for p16 (Santa Cruz Biotechnology inc, Europe) was diluted 1:200. Staining was performed in a Ventana Bench Mark Ultra staining machine (Ventana Medical Systems, Inc, Tucson, AZ, USA) according to the manufacturer’s recommendations.

-Scoring

Samples were scored regarding percentage of epithelial cells expressing p16 and intensity of staining. Proportion of p16 expressing cells was divided into six stages where 1=0–4%, 2=5–19%, 3=20–39%, 4=40–59%, 5=60–79% and 6=80–100%, and intensity in four stages where 0=negative, 1=weak, 2=intermediate and 3=strong staining. By multiplying percentage of p16 expressing cells with intensity for each sample a quick score (QS) ranging from 0 to 18 was obtained (16). The p16-stained slides were evaluated independently by four of the authors (KD, JO, RZ and ME). Depending on the result, the material is divided in to 3 groups. Those who did not express p16 (QS = 0), those with moderate expressions (QS 1-5) and those with high expressions (QS 6-18).

In cases of disagreement slides were discussed in a joint session until consensuses was reached.

-Statistical analysis

Statistical analysis was performed using IBM SPSS statistics v22. The non-parametric method Mann-Whitney was used. Significance level was set at *p*=0.05

## Results

A total of 103 slides from OLP and GLP lesions as well as 24 controls were evaluated.

Cases with QS> 0 were regarded as positive. Those with moderate expressions (QS 1-5) and those with strong expressions (QS 6-18).

In the OLP group all but two of the 79 samples were positive (97%), and the majority, 78.5%, showed a QS of 6-18. In the GLP group all cases were positive, and most samples, 66.5% had a QS of 6-18.

Among the oral controls 53% were negative, and none of the samples had a QS over 5. The genital controls had one negative case only and two cases with QS of 6-18.

In the group with a” moderate” QS, defined as a QS of 1-5, control samples dominated, 47% of the oral and 67% of the genital controls, compared to 19% and 33.5% respectively of the OLP and GLP cases.

**Figure 1:**
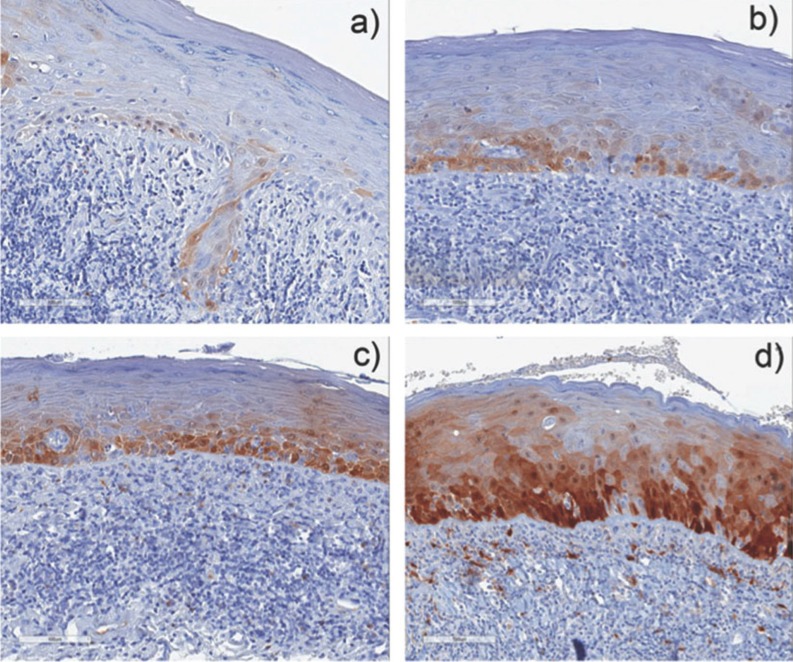
Representative IHC staining of p16 in OLP at X20 magnification. a) QS= 2, b) QS=6, c) QS= 12 and d) QS= 18.

Expression of p16 was significantly higher in OLP compared to control oral mucosa (OC) *p*=0.000, and the same pattern was seen in GLP compared with control genital mucosa (GC) *p*=0.002 (Fig. 1). The mean QS for the OLP group was 8.7 compared to 1.3 for controls, and 7.3 for GLP compared to 2.6 for genital controls.

**Figure 2:**
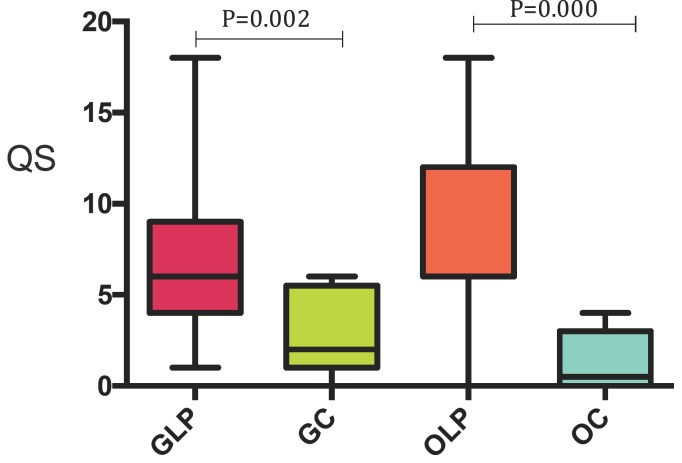
Box plot chart showing distribution of QS for OLP and GLP and corresponding control mucosa. (OC = Oral Control and GC= Genital Control).

Representative stainings are shown in Figure 2, and QS values in  Table 2.

**Table 2:**
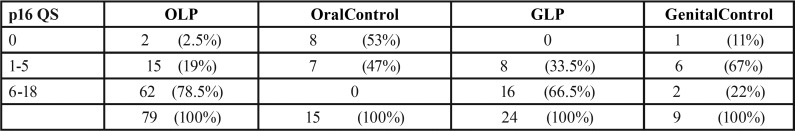
Results from QS of OLP, GLP and corresponding normal tissues.

## Discussion

This is, to our knowledge, the first study evaluating expression of p16 in genital LP in comparison to oral LP as well as normal corresponding mucosa. Malignant transformation of LP is a controversial issue and even if there are numerous publications no consensus has been reached (17). Even though GLP is not classified as a premalignant condition by WHO there are reports regarding malignant transformation of erosive GLP (18,19) 

Overexpression of p16 has been reported in different types of cancer including oral squamous cell carcinoma (4,10). Over the past decade different results have been published regarding the relationship between expression of p16 in normal mucosa, oral premalignant lesions and oral cancer (4,12,14).

Data from the present study showing p16 to be significantly highly expressed in both OLP and GLP, while expression was relatively low in healthy control samples is in accordance with some of the previous studies performed on OLP (4,12,13). In contrast, as far as we know, no data on p16 expression in genital LP is available.

p16 can by inhibiting the cdk4/cdk6 complex regulate the cell cycle and thus also gene expression, (20). High expression of p16 could therefore be an indication of cell cycle deregulation. p16 can in certain tumour types like tonsillar cancer act as a surrogate marker for high risk Human Papilloma Virus (HPV) (21), whereas no such correlation is seen in tongue cancer (11). A recent study by Montebugnoli et al also showed lack of association between p16 expression and HPV in OLP (22).

In LP lesions increased levels of TNF- α and IFN-γ are seen (23), and cytokines like interferons and TNF-α have a significant role in initiation of inflammation. A combination of IFN-γ and TNF- α further induces p16 and accordingly permanent growth arrest (4,12). Serum levels of TNF-α are known to be higher in OLP patients compared to healthy individuals (24), and TNF- α has also been seen to be overexpressed in basal keratinocytes in patients with OLP (25). The higher p16-expression seen in our LP-samples could thus be caused by the cytokine activity in OLP and GLP causing inflammatory progression and in turn protection from cancer development. The role of TNF is, however, controversial, where some studies have shown high concentrations of TNF- α to act as a tumour suppressor in animal models whereas low levels in contrast can induce cancer (26). In addition there are some evidence from animal models that TNF in high concentration is capable of killing tumour cells (27). IFN-γ another cytokine overexpressed in LP has several roles in initiating and maintaining inflammation and is involved in events associated with up regulation of p16 (28)

In oral squamous cell carcinoma high p16 expression has been correlated to better prognosis (10,29) and low expression to increased risk of relapse (30). The increased expression of p16 that we saw could thus exert a protection against malignant transformation. Data published by Natarajan et al presented a role for p16 in wound healing and senescence. p16 together with laminin 5 is involved in a hyper motility/growth arrest response when keratinocytes are exposed to a compromised basement membrane. (31) 

In conclusion, we show statistically significantly higher expression of p16 in OLP and GLP compared to normal oral and genital mucosa and suggest that this over-expression may act as a protection against malignant transformation. Furthermore, data indicate that lichen planus is one general disease establishing in different locations.
